# Influence of Disc Size on the Diagnostic Accuracy of Cirrus Spectral-Domain Optical Coherence Tomography in Glaucoma

**DOI:** 10.1155/2018/5692404

**Published:** 2018-04-11

**Authors:** Shino Sato, Kaori Ukegawa, Eri Nitta, Kazuyuki Hirooka

**Affiliations:** Department of Ophthalmology, Kagawa University Faculty of Medicine, 1750-1 Ikenobe, Miki, Kagawa 761-0793, Japan

## Abstract

**Purpose:**

To examine the influence of optic disc size on the diagnostic accuracy of optic nerve head (ONH) parameters determined by Cirrus spectral-domain optical coherence tomography (Cirrus HD-OCT).

**Methods:**

A total of 51 eyes of 51 normal participants and 71 eyes of 71 glaucoma patients were examined. ONH imaging was obtained by Cirrus HD-OCT. Sensitivity at a fixed 90% specificity along with the area under the receiver operating characteristic curve (AUC) for continuous parameters were analyzed. We also examined the coefficients of variation (CoV) for sensitivity estimates, as these have been used to test and quantify the influence of optic disc size on diagnostic accuracy. The influence of optic disc size on the glaucoma diagnosis was assessed by the likelihood ratio chi-square test.

**Results:**

Among the continuous parameters, the best diagnostic accuracy was seen for the average rim area, which had an AUC of 0.96. The most reliable factor across the disc size groups was the rim area (CoV, 2.8%). The diagnostic accuracy of the rim area did not appear to be influenced by optic disc size (*P* = 0.17).

**Conclusions:**

The high diagnostic accuracy of the rim area demonstrated by Cirrus HD-OCT for the quantitative assessment of the ONH was not significantly affected by disc size in this study.

## 1. Purpose

Primary open-angle glaucoma (POAG) is a chronic disease characterized by a progressive loss of retinal ganglion cells [1]. This cell loss leads to structural damage that includes a progressive regional or diffuse thinning of both the retinal nerve fiber layer (RNFL) and the neuroretinal rim within the optic nerve head (ONH), followed by functional loss, as shown by progressive visual field defects [2].

Optical coherence tomography (OCT) is a noninvasive technology that has been extensively used to evaluate many retinal and optic nerve diseases. OCT can be used to analyze and measure not only the peripapillary RNFL thickness [3–5] but also topographic parameters of the ONH, including the disc area, neuroretinal rim area, and the cup-to-disc ratio [6]. Since 2002, time-domain OCT (Stratus OCT, Carl Zeiss Meditec, Dublin, CA) has been used for ONH analysis. The recently validated spectral-domain technology, such as the Cirrus HD-OCT (version 5) (Carl Zeiss Meditec), has been shown to provide a better approach for identifying the retinal pigment epithelium (RPE) in the vicinity of the disc, thereby ensuring that the termination point of Bruch's membrane can be found [7, 8]. Moreover, software version 6.0 of the Cirrus HD-OCT system has led to a further significant improvement of the ONH segmentation quality.

The Heidelberg Retinal Tomograph (HRT; Heidelberg Engineering GmbH, Dossenheim, Germany) is the leading device for 3-dimensional quantitative studies and classification of the ONH shape [9, 10]. Subsequent studies have shown that the accuracy of the discrimination between normality and glaucoma is significantly influenced by both the ONH shape and size. Specifically, it has been demonstrated that larger optic discs are associated with lower specificities, while smaller discs are associated with lower sensitivities [11–14]. Therefore, these can potentially limit the role of quantitative ONH assessments during the clinical diagnostic process.

The purpose of the current study was to examine the influence of the ONH size on the diagnostic accuracy of the ONH quantitative assessment determined by the Cirrus HD-OCT.

## 2. Methods

A total of 71 glaucoma patients and 51 normal participants were enrolled in the study. All patients were examined at Kagawa University Hospital between August 2012 and November 2012. Among the patients, 27 had POAG while 44 had normal-tension glaucoma (NTG). After being provided with a detailed explanation of the study, all eligible participants signed an informed consent form in accordance with the principles embodied in the Declaration of Helsinki. The Institutional Review Board of the Kagawa University Hospital approved this study. The normal control group consisted of participants attending outpatient clinics, spouses and friends of the recruited patients, and volunteers from our hospital staff.

All participants underwent a complete ophthalmic examination. Tests performed included visual acuity testing with refraction, intraocular pressure (IOP) measurement, gonioscopy examination, and a dilated fundus examination with stereoscopic biomicroscopy of the optic nerve head conducted via slit lamp and indirect ophthalmoscopy. All participants were required to have a best-corrected visual acuity of 20/40 or better, a spherical error within a range between +4.0 and −6.0 diopters, a cylinder within ±2.0 diopters, and open angles (grade 3 and 4 according to Shaffer grading) in order to be included in the study. Subjects were excluded if they had a history of any kind of retinal pathology or neurologic disease or had previously undergone a retinal laser procedure or retinal surgery. One eye was randomly chosen for examination in each participant in the study. The enrolled normal controls were required to have a normal visual field with an IOP ≤ 21 mmHg and no history of retinal pathology. Eyes were defined as being glaucomatous if they exhibited structural glaucomatous changes that included a cup-to-disc ratio of ≥0.6, vertical cup-disc asymmetry between fellow eyes of ≥0.2, and narrowing of the neuroretinal rim, notches, localized pallor, or the presence of defects in the RNFL with glaucomatous VF loss in the corresponding hemifield. In order for the VF to be defined as glaucomatous, the glaucoma hemifield test (GHT) had to be outside of the normal limits, and after excluding the points on the edge of the field or those directly above and below the blind spot, it was necessary for at least three contiguous test points to be present within the same hemifield on the pattern deviation plot at *P* < 1%, with at least one at *P* < 0.5% [15]. The target condition under investigation was “definite glaucoma,” and the reference standard in this study was VF defect definition and structural glaucomatous change.

The severity of the visual field damage was assessed using the VF index (VFI). Details of the VFI calculation have been described elsewhere [16]. In brief, the VFI represents the percentage of normal age-corrected visual function, with this value intended for use in calculating the progression rates and staging of glaucomatous functional damage. Use of VFI to evaluate the rate of functional loss in glaucomatous eyes has been suggested to be less susceptible than the mean deviation to the effects of cataract or diffuse media opacities [17]. VFI values can range from 100% (normal visual field) to 0% (perimetrically blind field).

## 3. Cirrus HD-OCT Imaging

All eyes were scanned by the Cirrus HD-OCT system, which used software version 6.0. The ONH was centered on the live image, after which the centering and enhancement were optimized. Subsequently, the laser scanned a 6 × 6 mm area, which captured a cube of data that consisted of 200 A-scans from 200 linear B-scans (40,000 points) over approximately 1.5 s (27,000 A-scans). After first determining the optic disc, the Cirrus HD-OCT algorithms automatically placed a calculation circle of 3.46 mm in diameter evenly around the disc. Software version 6.0 calculated the ONH parameter results via the use of a fully automatic algorithm that defined both the disc and cup margins within the three-dimensional data cube. The present study performed global measurements that included the disc area, rim area, cup-to-disc ratio, horizontal cup-to-disc ratio, and cup volume as previously described [18]. All the scans had a signal strength of at least 6. Both VF testing and OCT measurements were performed during the same visit.

## 4. Statistical Analysis

Continuous data were described by mean values ± standard deviation (SD) while categorical data were described by frequency analysis. Differences between the control and glaucoma groups were assessed by an independent Student's *t*-test, and the chi-square test for categorical parameters. *P* < 0.05 was considered statistically significant. Sensitivities were compared between the parameters by choosing cutoff points that corresponded to a fixed 90% specificity. The influence of disc size on the diagnostic accuracy of the imaging devices under evaluation was assessed using the following method. The study population was first divided into 2 disc size groups based on the cutoff of the median value of the disc area [19]. Analyses were then performed in both groups, with the coefficient of variation (CoV) calculated for the sensitivity estimates using the following formula: CoV = [standard deviation of sensitivities across the disc size groups/mean of the sensitivities across the disc size groups]∗100. The influence of the optic disc size on the glaucoma diagnosis was subsequently assessed by each parameter of OCT and was assessed by likelihood ratio chi-square test. Statistical analyses were performed using SPSS version 19.0 (IBM, New York).

## 5. Results

**Figure 1:**
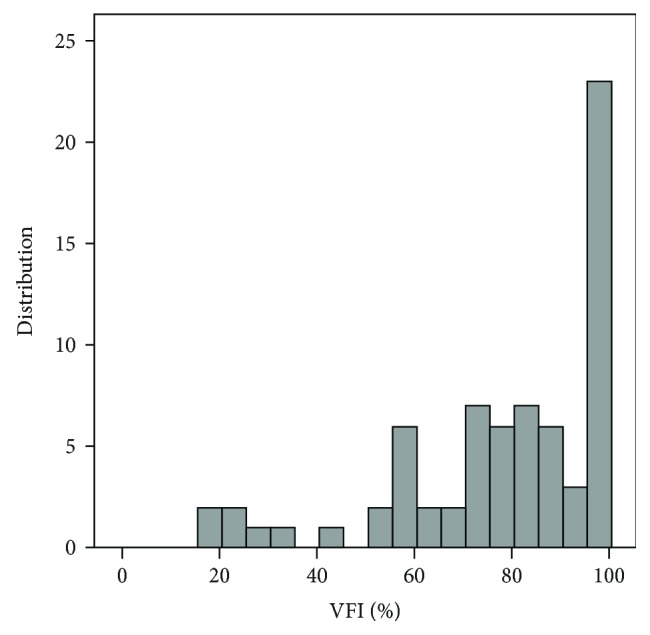
Distribution of disease severity, as measured by the VFI in the glaucoma group.

**Figure 2:**
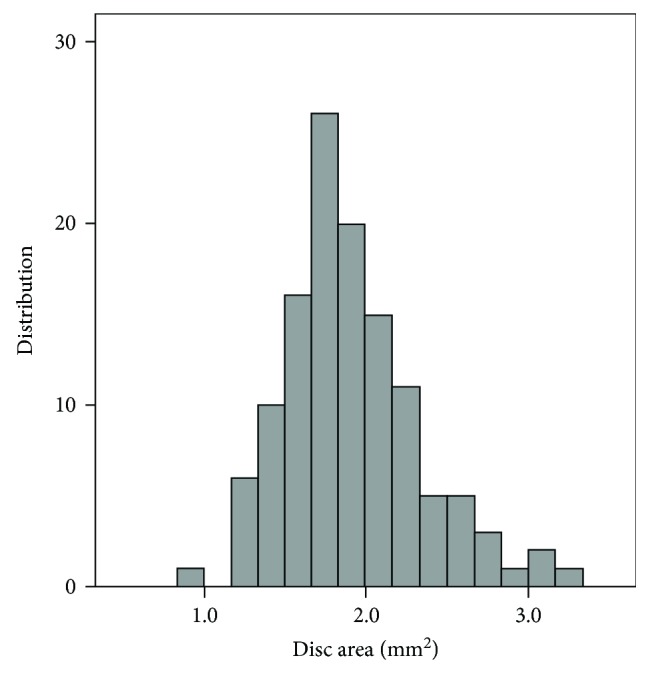
Distribution of optic disc size in the entire cohort.

Table 1 presents the basic characteristics of the diagnostic groups. Patients in the glaucoma group were significantly older than the participants in the normal group.  Figure 1 shows the distribution of the disease severity, which was based on the VFI in the glaucomatous eyes.  Figure 2 shows the distribution of the optic disc size in the entire cohort. The mean disc area was 1.91 ± 0.41 mm^2^ (median, 1.84).

Thirty-four glaucoma patients and 25 normal participants were in small disc group and 37 glaucoma patients and 26 normal participants were in large disc group (Table 2). The rim area provided a sensitivity of 92% at a specificity of 90%, which corresponded to a cutoff of 0.96 mm^2^. The variability of the diagnosis sensitivity across the disc size ranged from 2.8% to 48.9%, with the rim area parameter found to produce the most stable values among the small and large discs (sensitivity of 88% and 92% in the small and large discs, respectively; CoV = 2.8%). Sensitivity at fixed specificity was recovered from the receiver operating characteristic curve plot.

Glaucoma diagnosis using the rim area was not affected (*P* = 0.17) by the disc size, although the other parameters were associated with diagnosis (*P* < 0.05, Table 3).

There was no significant difference of disease severity between the large disc group (81.5 ± 21.1%) and the small disc group (75.7 ± 23.2%; *P* = 0.28).

## 6. Discussion

In the current study, we demonstrated that the optic disc size did not influence the accuracy of the Cirrus HD-OCT when determining the ONH rim area parameter. While the rim area was determined to be the most sensitive of the ONH parameters (sensitivity 92%), the cup volume, however, was found to be a poor diagnostic parameter. A previous study has shown that values above 80% for fixed specificities of 90% indicate very good discriminating capabilities between healthy and glaucomatous eyes [20].

Other previous studies [11–14] that used the Heidelberg Retina Tomograph (HRT, Heidelberg Engineering, Heidelberg, Germany) have shown that the size of the optic disc can significantly influence the diagnostic accuracy of a quantitative ONH assessment, with larger discs associated with lower specificities and smaller discs associated with lower sensitivities. Coops et al. previously reported that optic disc size had a significant effect on the HRT3 classification, with an estimated 21% increase in the odds of a positive glaucoma probability score (GPS) classification and a 15% positive Moorfields regression analysis (MRA) classification for each 0.1 mm^2^ increase in disc size [17]. The diagnostic accuracies for the HRT parameters, particularly the linear discriminant functions and the MRA, have been found to improve in conjunction with increasing disc sizes. It has been suggested that this may be due to difficulties in detecting the neuroretinal rim loss in a small disc as compared to a large disc [19].

In contrast to these previous findings, Medeiros et al. recently demonstrated that the sensitivities of the Cirrus HD-OCT RNFL parameters were not significantly influenced by the size of the optic disc [19]. Instead, they reported that larger discs were associated with higher sensitivities, and that these were responsible for significantly influencing the HRT diagnostic performance. It has also been reported that the disc size has no significant influence on the AUCs for any of the RTVue (Optovue, Fremont, CA) scanning protocols [21]. Additionally, the rim area has been shown to have very good sensitivities when above 80% for fixed specificities of 90% in both small and large discs. This demonstrates the excellent capabilities of this parameter for successfully discriminating between healthy and glaucomatous eyes. At the present time, it is unknown why HRT imaging is affected by disc size while OCT appears not to be affected. One possible explanation could be that there was a type II error associated with this study.

Since diagnostic accuracies are significantly influenced by disease severity [19, 21–23], it is important to adjust for the severity of diseases when comparing the effect of the disc size on the diagnostic accuracy of the imaging tests. In the current study, we found no significant differences for disease severity between the large and small disc groups.

One of the limitations of our current study was that the sample size in this study was modest, which could have led to relatively wide confidence limits for our estimates of the sensitivity. Also, we based the disc size in this study on the Cirrus HD-OCT disc area. By using the disc area from the same instrument, this could have potentially introduced bias for the other parameters that were being evaluated. Therefore, in order to determine more precise estimates of the diagnostic accuracy, larger studies will need to be undertaken in the future.

In conclusion, the results of this study indicate that Cirrus HD-OCT can be used to precisely determine the ONH rim area, with variations in the optic disc size having virtually no or only a minimal effect on the diagnostic accuracy.

## Figures and Tables

**Table 1:** Clinical characteristics in study participants.

	Normal	Glaucoma	*P* value
*N*	51	71	
Age (y)	61.0 ± 10.1	64.5 ± 11.7	0.09
Gender (M/F)	24/27	25/46	0.19
MD (dB)		−6.85 ± 7.66	
VFI (%)		78.4 ± 22.1	
Disc size (mm^2^)	1.89 ± 0.36	1.92 ± 0.45	0.64
Spherical equivalent (D)	−1.26 ± 1.98	−1.00 ± 2.30	0.51

M: male; F: female; MD: mean deviation; VFI: visual field index.

**Table 2:** Sensitivity at fixed specificity of optic nerve head measured by Cirrus HD-OCT in control and in glaucoma.

	All discs		Small discs		Large discs		
	Sensitivity at specificity > 90%	Cutoff	Sensitivity at specificity > 90%	Cutoff	Sensitivity at specificity > 90%	Cutoff	Sensitivity CoV (%)
Rim area	0.92	0.96 ^∗^	0.88	0.88^∗^	0.92	0.96 ^∗^	2.8
Average C/D	0.68	0.74	0.71	0.69	0.76	0.76	4.9
Vertical C/D	0.83	0.71	0.76	0.71	0.89	0.78	10.9
Cup volume	0.37	0.45^#^	0.5	0.33^#^	0.24	0.73^#^	48.9

CoV: coefficient of variation of sensitivity across small and large disc groups; ^∗^: mm^2^; ^#^: mm^3^.

**Table 3:** Factors associated with the diagnostic accuracy of disc size.

	*P* value
Rim area	0.17
Average C/D	<0.01
Vertical C/D	0.02
Cup volume	<0.01
